# Supplementary figures and images for: Visual scanning strategies in the cockpit are modulated by pilots’ expertise: A flight simulator study
Source: PLoS One. 2021 Feb 18;16(2):e0247061. doi: 10.1371/journal.pone.0247061 (PMC7891757; doi:10.1371/journal.pone.0247061)

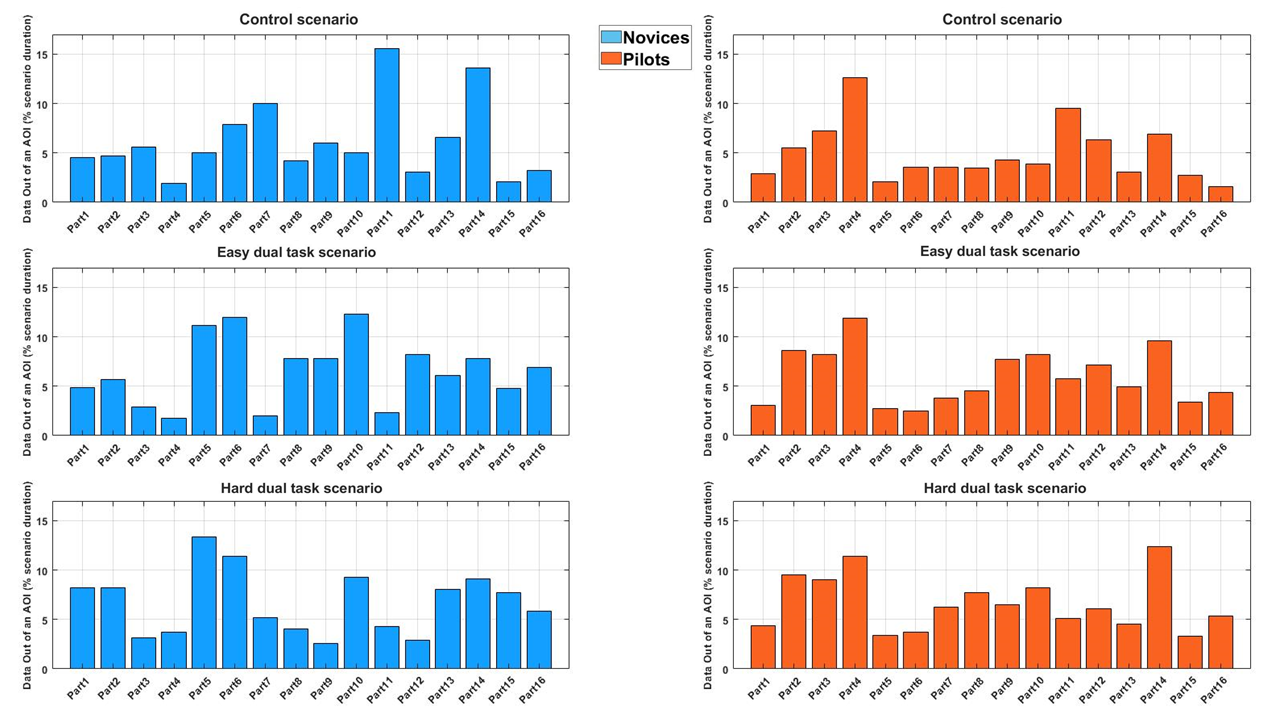

Supplement: S1 Fig — (TIF) [file pone.0247061.s001.tif]
